# Decreased circulatory levels of Vitamin D in Vitiligo: a meta-analysis^☆^^☆☆^

**DOI:** 10.1016/j.abd.2020.10.002

**Published:** 2021-03-24

**Authors:** Seshadri Reddy Varikasuvu, Sowjanya Aloori, Saurabh Varshney, Aparna Varma Bhongir

**Affiliations:** a Department of Biochemistry, All India Institute of Medical Sciences, Deoghar, India; b Department of Health Education, Telangana State Residential School & College, Choutuppal, India; c All India Institute of Medical Sciences, Deoghar, India; d Department of Biochemistry, All India Institute of Medical Sciences, Bibinagar, India

**Keywords:** Meta-analysis, Vitamin D, Vitiligo, 25-hydroxyvitamin D 2

## Abstract

**Background:**

The serum Vitamin D status in patients with vitiligo is ambiguous when compared to controls. A systematic review and updated meta-analysis were conducted to evaluate the association between Vitamin D and vitiligo.

**Methods:**

Relevant studies were identified by searching PubMed and other databases. The random effects model was used to obtain standardized mean differences and pooled correlation coefficients. Meta-regression and sub-group analyses were conducted to explore heterogeneity. The presence of publication bias and the study robustness were tested using funnel plot and sensitivity analyses, respectively.

**Results:**

This meta-analysis finally included 31 studies. Compared with controls, vitiligo patients showed significantly decreased serum Vitamin D levels (standardized mean difference = −1.03; p < 0.0001). The sub-group analysis showed that vitiligo patients with indoor/urban work had a significantly lower Vitamin D level when compared to their outdoor/rural counterparts (standardized mean differences = −0.45; p = 0.03). The sensitivity analysis indicated that no single study had a significant influence on the overall outcome, suggesting the robustness of this meta-analysis.

**Study limitations:**

Varied sample sizes and heterogeneous study populations from different countries are the limitations of this study. However, the between-study heterogeneity has been addressed by the random-effects model with meta-regression and sensitivity analyses.

**Conclusions:**

This meta-analysis showed significantly decreased Vitamin D level in vitiligo, and its association with indoor/outdoor type of work of vitiligo patients. This study highlights the need to assess Vitamin D status for improving its level in vitiligo.

## Introduction

Vitamin D (Vit-D) has pivotal involvements from bone structure to brain function and is also vital for healthy skin.^1^ Besides, it plays an important role in the immune system. Hypovitaminosis D has been known to be associated with the disorders of calcium and carbohydrate metabolism, cancer, cardiovascular, and several autoimmune diseases.^2^ Vitiligo is a skin disease known to have metabolic, cytotoxic, inflammatory, and autoimmune etiologies among several other factors.^3^, ^4^ The active form of Vit-D; 1.25-dihydroxyvitamin D3 (1.25(OH)D) has been reported to be associated with the pathogenesis of Vitiligo.^1^, ^2^, ^3^ As epidermis is affected in vitiligo, changes in the circulatory Vit-D levels could be evidenced.^2^, ^3^, ^4^

The available studies in the literature are inconsistent on Vit-D levels and Hypovitaminosis D is still controversial in vitiligo.^5^, ^6^, ^7^, ^8^, ^9^, ^10^, ^11^, ^12^, ^13^, ^14^, ^15^, ^16^, ^17^, ^18^, ^19^, ^20^, ^21^, ^22^, ^23^, ^24^, ^25^, ^26^, ^27^, ^28^, ^29^, ^30^, ^31^, ^32^, ^33^, ^34^ In this updated systematic review and meta-analysis on vitiligo and Vit-D levels (ViViD Study), the serum Vit-D levels and their association with variables like age, disease duration, affected Body Surface Area (BSA), and Vitiligo Activity and Severity Index (VASI) were studied in vitiligo patients as compared to controls. A subgroup meta-analysis was conducted to study Vit-D levels in relation to gender, skin type, Vitiligo type, disease activity, vitiligo history, autoimmune comorbidities, and indoor-outdoor job status of vitiligo patients.

## Material and methods

The criteria of Preferred Reporting Items for Systematic reviews and Meta-Analysis (PRISMA) were followed in the conduction and reporting of this study. The study protocol has been registered with PROSPERO having registration number: CRD42019142156 (https://www.crd.york.ac.uk/PROSPERO/display_record.php?RecordID=142156).

### Literature search strategy

The literature search was primarily conducted in the NCBI PubMed/MEDLINE database using both the MESH and text word search strategies. The following search string has been developed: ("vitamin D" (MeSH Terms) OR "vitamin D" (All Fields) OR "ergocalciferols" (MeSH Terms) OR "ergocalciferols" (All Fields)) AND ("vitiligo" (MeSH Terms) OR "vitiligo" (All Fields)). The other terms used for Vit-D were 25-Hydroxyvitamin D, 25-Hydroxycholecalciferol, calcidiol, 1.25-dihydroxyvitamin D3, and calcitriol. Also, the Google scholar, Cochrane library, Springer’s author mapper, science direct, clinical trial registries, and CINHAL databases were also searched for articles that reported circulatory Vit-D levels in vitiligo patients. Furthermore, bibliographies of published articles were manually hand-searched to identify additional studies. Two authors have independently performed the literature search, and any discrepancies were resolved upon discussion. Efforts have been made to obtain data, if any, from unpublished sources. When required, the corresponding authors of respective articles were contacted through e-mail to obtain data/clarification. All the literature searches were carried out prior to 5^th^ September 2019 with no time span specified, with and without humans set as a limit.

### Study selection criteria

The following criteria are followed for individual studies to be included in this meta-analysis: (1) papers published in a peer-reviewed journal; (2) provided original data; (3) study subjects were human with a description of vitiligo and controls; (4) studies where serum Vit-D levels in vitiligo patients were compared with controls; (5) provided method description for Vit-D estimation; (6) articles written in English/Chinese with clear data; (7) studies diagnosing vitiligo clinically by a dermatologist. The exclusion criteria were as follows: (1) studies with no control group; (2) reported on other skin diseases; (3) Vit-D levels were only shown by pictograms with no data; (4) reviews, commentaries, and letter to the editor article types. If any duplicate articles were found, only a recent report or all relevant information from either of the studies was included.

### Data extraction and quality assessment

The following information has been extracted from each of the included studies; first author names, country, study type and year of publication, number of vitiligo and control subjects, male and female subjects in each group, mean/median values of age, disease duration, means and SD metrics of Vit-D, specimen used for its measurement, method and units, vitiligo type and history, disease activity, skin types, autoimmune comorbidities, and other study characteristics. A quality score evaluation of included studies was done according to the Newcastle-Ottawa Scale (NOS).^35^ Quality assessment of study scores range from 0 to 9 points included three components: selection, comparability, and exposure.

### Statistical analysis

Meta-analyses and sub-group analyses were conducted if three or more studies were reporting the serum Vit-D levels in vitiligo and control groups. We calculated the Standardized Mean Difference (SMD) and its 95% Confidence Interval (CI) as a summary statistic for the difference of Vit-D level between vitiligo patients and controls. Furthermore, we have also performed a meta-analysis of correlations between Vit-D and other parameters in vitiligo. The effect size for SMD and pooled correlation coefficient values were presented as a Z-score. The Z-score with a p-value of <0.05 was considered statistically significant.

The between-study heterogeneity was examined by Cochrane’s Q statistic and expressed as the percentages of I^2^. A p-value of <0.05 or I^2^ statistic of >50% indicated significant heterogeneity. A random-effects model was used to compute SMD. The meta-regression analysis was performed to identify the potential source of heterogeneity using several covariates such as age, sample size, number of males/females, disease duration, Vit-D measurement method, country, and year of publication. The risk of publication bias was studied by funnel plot asymmetry with Begg’s and Egger’s tests. However, to avoid the risk of bias, general search terms were chosen for a guaranteed retrieval for the inclusion of articles reporting Vit-D levels as a secondary outcome. In the case of a p < 0.05 suggesting a statistical significance, the “trim-and-fill method” was used to correct that bias. To test the robustness of this meta-analysis, a one-study leave-out sensitivity analysis was performed. All comparisons were two-tailed, and all analyses were conducted using the Review Manager Software version 5.3 which presents SMD as Hedges g; the difference between the two means divided by the pooled standard deviation, with a correction for sample bias. The MedCalc version 16.2.0 was used for the meta-analysis of correlations. The meta-regression and publication bias were respectively performed using OpenMeta and Comprehensive meta-analysis, version 3 software.

## Results

**Figure 1:**
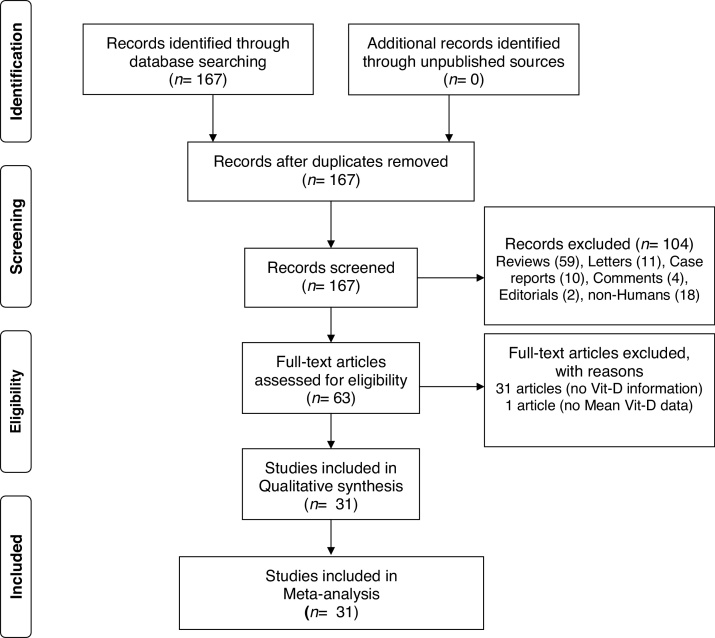
The PRISMA flow diagram of this meta-analysis.

A total of 167 papers were identified for Vit-D levels in vitiligo, of which 63 were retrieved for abstract and full-text review. Of these, a total of 31 articles did not deal with the circulatory Vit-D levels in vitiligo, therefore are excluded. In one more paper, no data has been reported and therefore is not included.^36^ In one study, though there is no control group, the study has been included for sub-group analysis of male-female differences in Vit-D levels in vitiligo.^37^ One study by Xu et al. has been considered twice as Vit-D levels were reported in two separate vitiligo and control groups studied at different time periods.^33^ Finally, a total of 31 articles were included in this meta-analysis.^5^, ^6^, ^7^, ^8^, ^9^, ^10^, ^11^, ^12^, ^13^, ^14^, ^15^, ^16^, ^17^, ^18^, ^19^, ^20^, ^21^, ^22^, ^23^, ^24^, ^25^, ^26^, ^27^, ^28^, ^29^, ^30^, ^31^, ^32^, ^33^, ^34^ The PRISMA flow diagram for this meta-analysis has been shown in Fig. 1. The detailed characteristics of the included studies and the NOS quality scores are presented in Table 1. With the obtained score range from 4 to 8, the overall quality of studies was medium to high. Of these included studies, 15 studies measured Vit-D by ELISA, 6 studies by chemiluminescence, 2 studies by RIA, one study by LCMS, and are not clear in 7 studies. While all the studies reported calcidiol levels, one study reported calcitriol concentrations.^5^ Except for two studies using plasma, all the studies used a serum for Vit-D measurement.^13^, ^30^ While most of the studies excluded previous treatment for vitiligo, oral vitamin D supplementation, and any topical or systemic treatment that affects vitamin D levels, it is unclear in some studies.^8^, ^12^, ^18^, ^28^ Studies where Narrowband Ultraviolet B (NB-UVB) treatment and/or vitamin D intervention were studied, only baseline vitamin D levels in vitiligo patients in comparison to controls were considered for meta-analysis.^14^, ^16^, ^20^, ^27^^,^^32^

**Table 1:** The study characteristics.

Study/ Country (study period) (REF)	Study type/SKT	Vitiligo				Controls		Matching	Vitamin D									NOS
		N (M/F)	Age	Type	DD	N (M/F)	Age (y)		Sample/Method	Units (change)	Vit-D	Vitiligo (n)			Controls (n)			
												Def	In-Suf	Suf	Def	In-Suf	Suf	
Aly et al., 2017/Egypt (Nov–Dec 2015)^5^	Cross-sectional/III, IV	30 (9/21)	30.23	Nonsegmental; Vulgaris Acrofacial Focal	5.83y	40 (19/21)	35.38	Age, sex	Serum/ELISA	ng/mL (SL)	(<10, 10–30, ≥30 ng/mL)	5	25	0	0	34	6	8
Amon et al., 2018/US (Jul 2015–Jun 2016)^6^	Retrospective/NC	113 (57/56)	38.2	NC	NA	71 (17/54)	49.2	NC	Serum/ELISA	ng/mL (SL)	(≤20 ng/mL, NA, NA)	46	NA	NA	1	NA	NA	5
Beheshti et al., 2014/Iran (Mar–Jun 2012)^37^	Cross-sectional/III, IV	100 (42/58)	28.7	Vulgaris	5y	No control group			Serum/EIA	nmoL/L	(<25, 25–47, 47.7–144 nmoL/L)	NA	NA	NA	NA	NA	NA	5
Cerman et al., 2014/Turkey (Nov 2012–Mar 2013)^7^	Cross-sectional/NC	44 (27/17)	33.64	NA	60.55m	58 (34/24)	32.55	Age, sex, BMI	Serum/LCMS	ng/mL (SL)	(≤20 ng/mL, NA, NA)	31	NA	NA	19	NA	NA	8
Deepthi & Geetharani 2018/India (Dec16–May 17)^8^	Dissertation/III, IV	120 (59/61)	37.6	Segmental, Vulgaris, universal, focal, acrofacial	≤5 to ≥21 y	60 (29/31)	36.2	Age, sex	Serum/ELISA	ng/mL (SL)	(≤20, 20–30, >30 ng/mL)	70	28	22	22	12	26	6
Doss et al., 2015/Egypt (Jun–Sep 2013)^9^	Case-control/ IV	30 (20/10)	32.5	Universal Generalized Localized	58.4 m	30 (16/14)	28.0	Age, sex	Serum/ELISA	ng/mL (SL)	(≤20, 20–30, ≥30 ng/mL)	8	12	10	0	1	29	7
El-Hanbuli et al., 2018/Egypt (Jun–Sep 2016)^10^	Case-control/ IV	30 (16/14)	29.53	NA	14.87 m	30 (16/14)	29.53	Age, sex, skin type	Serum/ELISA	ng/mL (SL)	(<12, 12–20, >20 ng/mL)	20	8	2	0	0	30	7
Farag et al., 2018/Egypt (Mar–Jun 2016)^11^	Case-control/III, IV	50 (18/32)	28.76	Acrofacial Generalized	3.5y	25 (10/15)	28.16	Age, sex, skin type	Serum/ELISA	nmoL/L (SL)	(<25, 25–74, ≥75 nmoL/L)	40	10	0	0	23	2	6
Firjani 2019/Egypt^12^	Case-control/NC	21	NA	NA	NA	21	NA	Age, sex	Serum/NC	ng/mL (SL)	NA	NA	NA	NA	NA	NA	NA	4
Hassan et al., 2019/India^13^	Case-control/NC	100 (39/61)	28.66	NC	3.81y	100 (40/60)	NA	Age, sex	Plasma/CLIA	ng/mL (SL)	(<20, 20–30, ≥30 ng/mL)	75	15	10	19	35	46	7
Ibrahim et al., 2018/Egypt (Dec 2015–Dec 2016)^14^	Case-control/ III, IV	80 (40/40)	34.27	NC	NA	20 (10/10)	35.20	Age, sex	Serum/ELISA	nmoL/L (SL)	(<10, 10–29, >30 ng/mL)	NA	NA	NA	NA	NA	NA	7
Karagün et al., 2016/Turkey (Dec 2013–Mar 2014)^15^	Case-control/ NC	50 (28/22)	30.96	Generalized	2–25y	47 (30/17)	31.45	Age, sex, skin type	Serum/NC	ng/mL (ND)	NA	NA	NA	NA	NA	NA	NA	6
Khodair et al., 2019/Egypt (Mar–Jun 2018)^16^	Cross-sectional/II–V	30 (6/24)	30.7	Localized Generalized	4.5 y	30 (10/20)	30.3	Age, sex	Serum/ELISA	ng/mL (SL)	NA	0	22	8	0	11	19	6
Khurrum et al., 2016/Saudi Arabia (Jan 2011–Dec 2012)^17^	Case-control/NC	150 (90/60)	30.6	Generalized Acral Focal	8m	150 (76/74)	30.7	Age, sex	Serum/CLIA	ng/mL (ND)	(<10, 10–30, ≥30 ng/mL)	137	0	13	136	0	14	8
Liu et al., 2014/China^18^	Case-control/NC	86	19–56	NA	NC	30	19–56	Age, sex	Serum/ELISA	nmoL/L (ND)	(<25, 25–47.6, 47.4–250 nmoL/L)	32	44	10	5	19	6	6
Ochoa-Ramírez et al., 2019/Mexico^19^	Case-control/NC	173 (81/92)	18–45	Disseminated (generalized, universal), Localized (focal, acrofacial).	NA	184	NA	Age, sex	Serum/ELISA	ng/mL (SL)	NA	NA	NA	NA	NA	NA	NA	6
Omidian & Asadian 2018/Iran (Apr 2015–Mar 2016)^20^	Clinical trial/NC	30 (12/18)	36.93	Generalized	NA	30 (11/19)	32.03	Age, sex	Serum/ECL	nmoL/L (SL)	(≤10, 10–29, 30–100 nmoL/L)	14	13	3	18	10	2	7
Pillai & Dinachandran 2019/India (Jan–Apr 2018)^21^	Case-control/NC	50 (27/23)	30.96	Generalized	2–25y	50 (30/20)	31.45	Age, sex, skin type	Serum/NC	ng/mL (ND)	NA	NA	NA	NA	NA	NA	NA	7
Prakash & Karthikeyan 2017/India (Oct 2014–May 2016)^22^	Case-control/V	45 (26/19)	43.78	Vulgaris Acrofacial Focal	51.04 m	45	NA	Age, sex	Serum/ELISA	ng/mL (ND)	(<20, 20–30, >30 ng/mL)	23	22	0	21	21	3	6
Ramalingam & Tang 2018/ Malaysia (Jul–Dec 2014)^23^	Cross-sectional/I–IV	93 (46/47)	40.43	Non-segmental	3y	87 (43/44)	39.95	Age, sex, race, BMI	Serum/NC	ng/mL (ND)	(≤20, 20–30, >30 ng/mL)	37	29	27	42	23	22	7
Saleh et al., 2013/Egypt (Apr–Jun 2011)^24^	Case-control/III–IV	40 (18/22)	34.1	Nonsegmental	3.5 y	40 (18/22)	34.2	Age, sex, skin type, Vit-D intake	Serum/RIA	ng/mL (SL)	(≤20, 20–30, ≥30 ng/mL)	39	0	1	5	2	33	7
Saniee et al., 2019/Iran (Spring and summer 2017)^25^	case control/III–IV	98 (50/48)	30.06	NC	34.65m	98 (53/45)	29.45	Age, sex	Serum/ELISA	ng/mL (ND)	(<10, 10–30, >30 ng/mL)	17	53	28	17	46	35	6
Sanjay & Raval 2019/India (1 yr period)^26^	Cross sectional/NC	25 (15/10)	23.0	Focal Generalized Acrofacial Mucosal	NA	25 (16/9)	24.12	Age, sex	Serum/ECL	ng/mL (ND)	NA	NA	NA	NA	NA	NA	NA	4
Sehrawat et al., 2014/India^27^	Clinical study/IV–V	30 (10/20)	31.33	Generalized	10.47y	30 (10/20)	NA	Age, sex	Serum/ELISA	nmoL/L (SL)	(<25, 25–74, 75–250 nmoL/L)	24	6	0	3	27	0	6
Shalaby et al., 2017/Egypt (Dec 2014–Jun 2016)^34^	Case-control/NC	40 (30/10)	28.70	Non-segmental	4.7y	40 (25/15)	32.95	Age, sex	Serum/ELISA	nmoL/L (SL)	(<10, 10–30, >30 ng/mL)	8	22	10	0	4	36	6
Sobeih et al., 2016/Egypt (Jul–Dec 2013)^28^	Case-control/ III–V	75	31.5	Vulgaris Acrofacial Segmental	5.5 y	75	NA	Age, sex	Serum/ELISA	ng/mL (SL)	NA	NA	NA	NA	NA	NA	NA	5
Srirama 2016/India (4 m period)^29^	Case-control/NC	30 (11/19)	43.9	Clinical	2–5y	30 (11/19)	49.8	Age, sex	Serum/CLIA	ng/mL (SL)	(<20, 20–30, >30 ng/mL)	NA	NA	NA	NA	NA	NA	5
Takci et al., 2015/Turkey (Winter: Nov 2011–Feb 2012)^30^	Case-control/II, III	44 (24/20)	34.5	Vulgaris Generalized Localized	38.8m	43 (10/33)	33.0	Age, sex, skin type	Plasma/RIA	ng/mL (SL)	(<20, 20–30, >30 ng/mL)	32	NA	NA	13	NA	NA	7
Ustun et al., 2014/Turkey (b/w 2010–2011)^31^	Cross-sectional/II, III	25 (13/12)	33.9	Vulgaris	5.36y	41 (20/21)	34.7	Age, sex, skin type	Serum/CLIA	ng/mL (ND)	(<20, 21–29, 30–100 ng/mL)	20	5	0	34	6	1	8
Watabe et al., 2018/Japan (Sep 2015–Jan 2016)^32^	RCT/III, IV	45	50.89	Vulgaris	NC	45	Age matched	Age	Serum/ NC	ng/mL (SL)	(<20, 20–30, >30 ng/mL)	30	12	3	17	20	8	8
Xu et al., 2012/China (Mar–May 2011)^33^	Case-control/NC	171 (80/91)	34.1	Segmental, Non-segmental; generalized, universal, focal, halo nevi	NC	93	34.6	Age, sex	Serum/NC	nmol/L (ND)	(<25, 25–75, >75 nmoL/L)	57	114	0	57	36	0	7
Xu et al., 2012/China (Sep–Oct 2010)^33^	Case-control/NC	30	NA	Segmental, Non-segmental; generalized, universal, focal, halo nevi	NC	20	NA	Age, sex	Serum/NC	nmol/L (ND)	(<25, 25–75, >75 nmoL/L)	3	27	0	2	18	0	7

NC, Not Clear; NA, Not Available; ND, No Difference; NOS, Newcastle-Ottawa Scale; SL, Significantly Lower; DD, Disease Duration; SKT, Fitzpatrick Skin Types; y, years; M, Male; F, Female; n, Sample size; Def, Deficiency; In-Suf, Insufficiency; Suf, Sufficiency.

### Circulatory Vit-D level in vitiligo compared with controls

**Figure 2:**
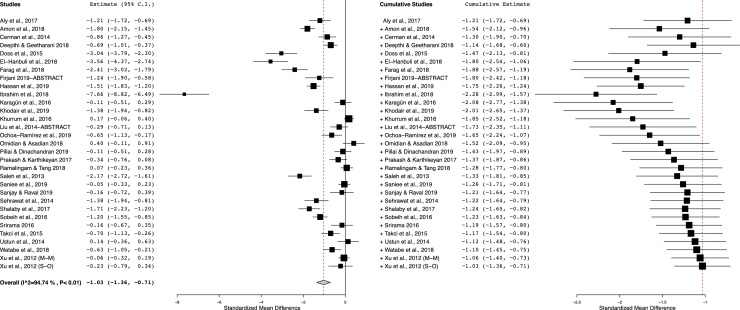
The forest plot and cumulative meta-analysis of included studies.

As shown in Fig. 2, vitiligo patients had decreased Vit-D levels compared with controls. With a significant between-study heterogeneity (I^2^ = 95%; p < 0.00001), the pooled SMD (95% CI) obtained with random-effects model was −1.03 (−1.35 to −0.71). This result suggests a significant difference in mean concentrations of Vit-D between vitiligo patients and controls, and the overall effect size for the obtained mean difference corrected for standard deviations and sample bias was statistically significant (Z = 6.22, p = 0.00001).

### Sub-group and meta-regression analyses

The results of subgroup meta-analysis based on country, Vit-D measurement method, vitiligo type, gender, skin type, vitiligo history, autoimmune comorbidities/history, disease activity (progressive/active or stable), job status (indoor or outdoor), and journal indexing (PubMed/Non-PubMed) were presented in Table 2. The pooled SMD was statistically significant in the subgroups of ‘Egypt’, ‘non-Egypt’, ‘ELISA’, ‘indoor vs. outdoor’, ‘PubMed’, and ‘Non-PubMed’. The statistical significance was not achieved in all other subgroups including different vitiligo types, skin type, disease activity, vitiligo history, AIC, and between male and female gender in vitiligo patients, which is suggestive of a possible source of heterogeneity. Among the several covariates used in meta-regression (Table 3), the variable ‘country’ produced a statistically significant result (coefficient = 0.19; p = 0.01) indicative of a significant source of heterogeneity among the included studies. The results of correlation meta-analysis presented in Table 4, showed no significant associations of Vit-D with age, disease duration, BSA and VASI.

**Table 2:** The subgroup analysis.

Subgroup	N of studies	I^2^ (%)	SMD	95% CI	Overall effect (Z)	p-value
Overall	31	95	−1.03	−1.35; −0.71	6.22	<0.00001
Country						
Egypt	10	94	−2.46	−3.21; −1.70	6.39	<0.00001
Non-Egypt	21	90	−0.43	−0.69; −0.17	3.22	0.001
Germany	1	NA	−1.80	−2.15; −1.45	10.09	<0.00001
China	3	0	−0.14	−0.34; 0.06	1.34	0.18
India	7	88	−0.63	−1.08; −0.17	2.70	0.007
Iran	2	56	0.12	−0.30; 0.55	0.56	0.57
Japan	1	NA	−0.63	−1.05; −0.20	2.91	0.004
Malaysia	1	NA	0.07	−0.23; 0.36	0.45	0.65
Mexico	1	NA	0.65	−1.13; −0.17	2.64	0.008
Saudi Arabia	1	NA	0.17	−0.06; 0.40	1.47	0.14
Turkey	4	77	−0.39	−0.84; 0.06	1.71	0.09
Method						
ELISA	15	95	−1.72	−2.27; −1.16	6.04	<0.00001
Non-ELISA	9	94	−0.54	−1.09; 0.02	1.91	0.06
CL	6	94	−0.20	−0.86; 0.46	0.59	0.56
RIA	2	94	−1.42	−2.86; 0.02	1.93	0.05
LCMS	1	NA	−0.86	−1.27; −0.45	4.11	<0.0001
NA/NC	7	66	−0.26	−0.52; −0.00	1.98	0.05
Vitiligo						
Type A vs. B	4	10	−0.08	−0.41; 0.25	0.48	0.63
Type A vs. C	5	37	0.37	−0.11; 0.86	1.51	0.13
Type B vs. C	3	22	0.22	−0.53; 0.96	0.57	0.57
Male vs. Female	11	80	0.24	−0.09; 0.56	1.43	0.15
Active vs. Stable	4	41	0.18	−0.21; 0.57	0.92	0.36
SKT-III vs. IV	4	48	−0.20	−0.64; 0.24	0.91	0.36
VFH-Positive vs. Negative	5	26	0.15	−0.16; 0.46	0.93	0.35
AIC-Positive vs. Negative	5	64	0.04	−0.48; 0.56	0.15	0.88
Indoor vs. Outdoor	3	68	−0.57	−1.15; 0.01	1.94	0.05
Indoor vs. Outdoor & U/R	4	60	−0.45	−0.86; −0.04	2.17	0.03
Journal Article Indexed						
PubMed/MEDLINE	18	96	−1.37	−1.86; −0.88	5.47	<0.00001
Non-PubMed/MEDLINE	13	90	−0.59	−0.96; −0.23	3.16	0.002

SMD, Standardized Mean Difference; CI, Confidence Interval; U/R, Urban or Rural; SKT, Fitzpatrick Skin Type; NA/NC, Not Available/Not Clear; ELISA, Enzyme-Linked Immunosorbent Assay; RIA, Radio Immune Assay; CL, Chemileuminiscence; LCMS, Liquid Chromatography-Mass Spectrometry; I^2^, Heterogeneity; Type A, Vulgaris/generalized/disseminated; Type B, Acrofacial; Type C, Focal/localized.

**Table 3:** The meta-regression analysis.

Variables	Coefficients	95% CI	SE	p-value
Total n	0.004	−0.003; 0.012	0.004	0.253
Vitiligo n	0.003	−0.009; 0.016	0.006	0.612
Controls n	0.015	−0.001; 0.031	0.008	0.059
Vitiligo-Male	0.009	−0.015; 0.034	0.013	0.468
Vitiligo-Female	0.007	−0.018; 0.032	0.013	0.593
Vitiligo-M/F Ratio	0.030	−0.073; 0.134	0.053	0.566
Controls -Male	0.038	0.001; 0.075	0.019	0.044
Controls -Female	0.031	−0.007; 0.068	0.019	0.107
Controls -M/F Ratio	0.030	−0.073; 0.134	0.053	0.566
Vitiligo-Age	0.042	−0.056; 0.140	0.050	0.405
Controls-Age	0.012	−0.105; 0.130	0.060	0.837
Disease duration	−0.021	−0.261; 0.219	0.122	0.863
Year	−0.086	−0.313; 0.140	0.116	0.455
Country	0.194	0.041; 0.347	0.078	0.013
Method	−0.450	−1.206; 0.306	0.386	0.243
NOS	−0.166	−0.756; 0.425	0.301	0.583

**Table 4:** Meta-analysis of correlations.

Vit-D correlation with	Studies	n	I^2^ (%)	r	95% CI	Z	p
Age	6	317	86.92	−0.144	−0.438–0.178	−0.874	0.38
DD	7	342	4.97	0.061	−0.052–0.173	1.057	0.29
BSA	4	202	0.00	0.135	−0.006–0.271	1.876	0.06
VASI	5	220	82.68	−0.197	−0.494–0.142	−1.143	0.25

Age, Age of Vitiligo patients in years; DD, Disease duration of Vitiligo; BSA, affected Body Surface Area; VASI, Vitiligo Area and Severity Index; I^2^, Inconsistency; r, pooled correlation coefficient; CI, Confidence Interval.

### Publication bias and sensitivity analysis

**Figure 3:**
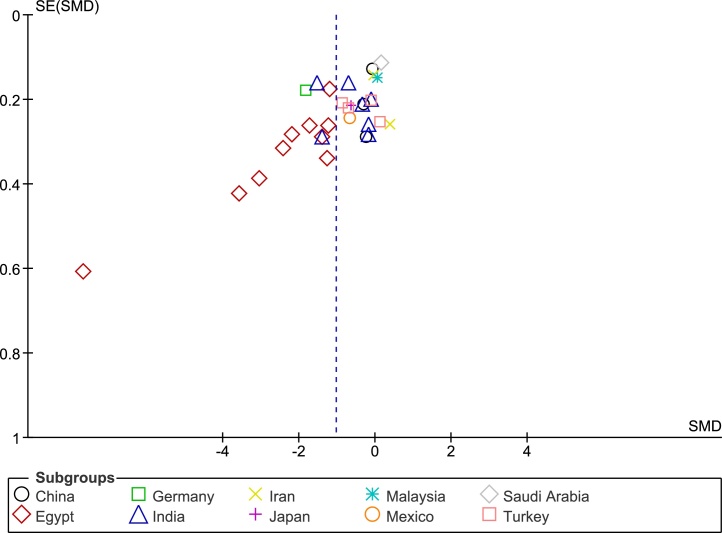
The funnel plot of publication bias.

**Figure 4:**
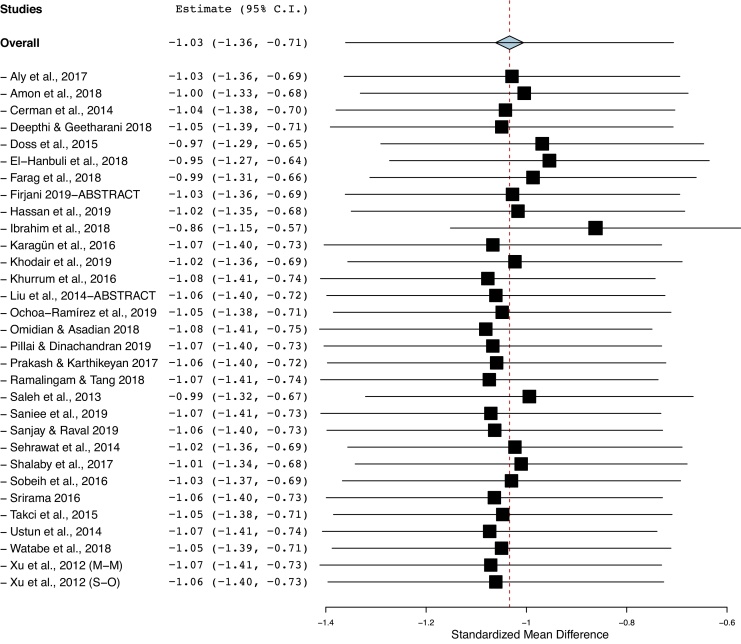
A one study leave-out sensitivity analysis.

The funnel plot analysis with Begg’s correlation (p < 0.001) and Egger’s regression tests (p < 0.001) indicate there might be a publication bias among the included studies (Fig. 3). However, it is unlikely that publication bias posed a significant effect on this meta-analysis as correcting that bias with the ‘trim-and-fill method’ substantiated our original results. With seven studies to be trimmed, the corrected values (SMD = −1.41, 95% CI: −1.81 to −1.00) still indicated significantly lower Vit-D levels in vitiligo as compared to controls. Further, a one-study-leave-out sensitivity analysis showed that no single study had significantly influenced the combined effect size. The combined SMD obtained was stable and remained statistically significant after leaving-out any particular study in the sensitivity analysis (Fig. 4).

## Discussion

Available literature shows contrasting results with regard to Vit-D levels in vitiligo.^5^, ^6^, ^7^, ^8^, ^9^, ^10^, ^11^, ^12^, ^13^, ^14^, ^15^, ^16^, ^17^, ^18^, ^19^, ^20^, ^21^, ^22^, ^23^, ^24^, ^25^, ^26^, ^27^, ^28^, ^29^, ^30^, ^31^, ^32^, ^33^, ^34^ To our best knowledge, the two systematic reviews available on the topic are not updated and are limited with respect to the number of included studies, sub-group, and meta-regression analyses.^2^, ^3^ Further, in one of these two studies, the data extraction with regard to an included study was wrong.^2^, ^33^ And, in the other study, both the adult and child vitiligo were included in the meta-analysis.^3^ In this updated study, we performed a meta-analysis including 1840 vitiligo patients and 1539 controls from 31 reports. The result of our meta-analysis shows that circulatory Vit-D concentration is significantly lower among vitiligo patients as compared to controls (SMD = −1.03, p < 0.00001).

The active form of Vit-D and Vitamin D Receptor (VDR) mediates several important biological functions such as calcium and phosphate metabolism, bone formation, growth, and differentiation. Studies have indicated that VDR polymorphisms are associated with Vit-D levels and exhibit a genetic predisposition for vitiligo.^3^, ^36^ Though the etiopathogenesis of vitiligo is not completely clear, Vit-D is known to mediate immune regulation and immune diseases in addition to diabetes and cardiovascular diseases among others.^1^, ^2^, ^3^ It is also known that vitiligo is associated with autoimmune comorbidities and therefore altered circulatory Vit-D levels may be associated with vitiligo patients. The several factors which can influence the circulatory Vit-D level include vitiligo type, skin type, disease activity, vitiligo history, autoimmune comorbidities, age, indoor-outdoor job status, sun exposure, seasonal variations, and Vit-D measurement methods. In one cohort study, low Vit-D levels were reported to be associated with autoimmune comorbidities (Odds Ratio = 10.00), but not with age, gender, race/ethnicity, family history of vitiligo or autoimmune disease, new-onset disease, or affected BSA.^38^

Our sub-group analysis produced interesting results. As there were ten studies from Egypt, maximum for any other country, we performed subgroup meta-analysis based on Egypt and non-Egypt studies (21 studies). In both cases, statistical significance was retained for a decreased Vit-D level in vitiligo. However, when non-Egypt countries were further stratified, the subgroup India (7 studies) showed a statistical significance, whereas the sub-groups of studies from China (3 studies) and Turkey (4 studies) did not produce a statistically significant difference in Vit-D levels between their respective vitiligo and control groups (Table 2). This is suggestive of a between-study heterogeneity due to the ‘country’ of the included studies and the circulatory Vit-D levels may vary among countries because of different latitudes, genetic and food factors.^2^, ^3^ This is further substantiated by our meta-regression analysis indicating ‘country’ as a significant source of heterogeneity (Table 3). No significant differences were noted when Vit-D levels were compared between different types of non-segmental vitiligo, presence or absence of vitiligo history, and autoimmune comorbidities. Though the female and skin type IV vitiligo patients showed lower Vit-D levels, no statistical significance was achieved when compared to males and skin type III, respectively. The disease activity appears to have some effect with stable vitiligo showing lower Vit-D levels as compared to active vitiligo, no significant p-value was found (Table 2). Also, there was no significant influence from age, gender, sample size, year of publication, and disease duration was observed in the meta-regression analysis. Accordingly, the meta-analysis of correlation coefficients also yielded no statistically significant associations of Vit-D with age, disease duration, BSA, and VASI score (Table 4).

It is noteworthy that Vit-D levels were significantly lower in indoor/urban vitiligo patients when compared to their outdoor/rural counterparts (Table 2). This clearly suggests less outdoor activity is associated with decreased Vit-D levels in vitiligo. It indicates that the environment and hence the intensity of sun exposure may influence Vit-D levels. Although we could extract and summarize in Table 1, the different seasons/time frames during which the included studies were conducted and Vit-D measurements were made, data reported were insufficient to conduct a meta-analysis on the seasonal influence and associated Vit-D variations in vitiligo.

When a separate meta-analysis was performed based on ELISA (15 studies) and non-ELISA studies (9 studies), the statistical significance was only retained in a group of studies using ELISA, while the non-ELISA methodology (chemiluminescence in particular) is an important factor contributing to the heterogeneity among studies. However, in meta-regression (24 studies), the ‘method’ used as a covariate did not show a statistically significant value. Considering the PubMed/MEDLINE indexed papers are of good quality with credible information, we repeated a separate meta-analysis on PubMed (18 reports) and non-PubMed studies (13 reports), and results retained statistical significance in either case (Table 2).

Considering the increased risk of vitiligo and the risk of autoimmune comorbidities associated with decreased Vit-D status, our meta-analysis and previous studies point towards the need for maintaining sufficient Vit-D levels by phototherapy with NB-UVB and/or Vit-D supplementation.^39^, ^40^, ^41^ Further, antioxidant supplementation in combination with phototherapy has also been studied in vitiligo.^42^, ^43^ One recent study provides a piece of novel evidence on a significant positive association of sufficient Vit-D levels with the stability of the disease and a satisfactory repigmentation process in adult vitiligo patients.^41^ This previous evidence and this meta-analysis also strengthen the need to assess vitamin D status in vitiligo. Considering the therapeutic effectiveness of Vit-D supplementation and a reported correlation between sufficient Vit-D levels and the vitiligo disease course is promising, more randomized controlled trials are further needed on the role of Vit-D supplementation.^40^, ^41^ This also opens up the scope for a future meta-analysis of RCTs on the effectiveness of Vit-D supplementation in vitiligo.

Our study limitations include the between-study heterogeneity which could be attributable to heterogeneous study populations from different countries, different sample sizes, and differences in methodologies, varied environmental, genetic, dietary factors, and lifestyle habits, and other inherent limitations of case-control/observational study designs. However, as heterogeneity could not be completely eliminated in a meta-analysis, this has been addressed by applying the random-effects model for estimating the overall effect-size and by a cumulative and one-study leave out a sensitivity analysis to study whether any individual study would significantly affect the obtained overall effect-size. A one-study-removed sensitivity analysis revealed that no single study/observation had significantly influenced the pooled overall effect size. Therefore, our results could be considered robust. Further, all the included studies were matched for confounders like age and sex.

In conclusion, our meta-analysis indicates decreased Vit-D status in vitiligo patients as compared to controls. These results of our meta-analysis highlight the need to assess Vit-D status for improving its level in vitiligo. Further studies on the role of Vit-D with vitiligo pathogenesis and severity are needed in the future.

## Financial support

None declared.

## Authors’ contributions

Seshadri Reddy Varikasuvu: Designed the study; conducted literature search; performed analysis; wrote the manuscript; read and approved the final manuscript.

Sowjanya Aloori: Participated in literature search; selection criteria; data collection and revisions; read and approved the final manuscript.

Saurabh Varshney: Participated in literature search; selection criteria; data collection and revisions; read and approved the final manuscript.

Aparna Varma Bhongir: Assisted in literature search and revised the manuscript; read and approved the final manuscript.

## Conflicts of interest

None declared.
