# Erratum on “Decreased circulatory levels of Vitamin D in Vitiligo: a meta-analysis”

**DOI:** 10.1016/j.abd.2021.09.001

**Published:** 2021-11-09

**Authors:** Seshadri Reddy Varikasuvu, Sowjanya Aloori, Saurabh Varshney, Aparna Varma Bhongir

**Affiliations:** a Department of Biochemistry, All India Institute of Medical Sciences, Deoghar, India; b Department of Health Education, Telangana State Residential School & College, Choutuppal, India; c All India Institute of Medical Sciences, Deoghar, India; d Department of Biochemistry, All India Institute of Medical Sciences, Bibinagar, India

In the article “Decreased circulatory levels of Vitamin D in Vitiligo: a meta-analysis”, published in An Bras Dermatol. 2021;96(3):284-94, DOI of article original https://doi.org/10.1016/j.abd.2020.10.002, please consider the correct title as: “Decreased circulatory levels of Vitamin D in Vitiligo (ViViD Study): a meta-analysis”.

